# 2-[3-Acetyl-5-(2-chloro-3-pyrid­yl)-2-methyl-2,3-dihydro-1,3,4-oxadiazol-2-yl]-4-fluoro­phenyl acetate

**DOI:** 10.1107/S1600536809015323

**Published:** 2009-04-30

**Authors:** Quan Qin, Li Juan Xu, Li Fang Pan, Shui Qing Chen, Qing Bao Song

**Affiliations:** a The State Key Laboratory Breeding Base of Green Chemistry–Synthesis Technology, College of Chemical Engineering and Materials Science, Zhejiang University of Technology, Hangzhou 310014, People’s Republic of China

## Abstract

In the title compound, C_18_H_15_ClFN_3_O_4_, the dihedral angle between the substituted pyridine ring and the oxadiazo­line ring is 9.73 (19)° and the acyl group is coplanar with the oxadiazo­line ring [O—C—N—C torsion angle = −2.1 (3)°]. Furthermore, the substituted benzene ring is almost orthogonal with the oxadiazo­line ring, the dihedral angle between them being 87.56 (18)°.

## Related literature

For background to 1,3,4-oxadiazo­line derivatives and related structures, see: Song *et al.* (2006*a* ▶,*b* ▶); Pan *et al.* (2007 ▶). For the pharmacological properties of 2,5-disubstituted 1,3,4-oxa­diazo­lines, see: Chimirri *et al.* (1994 ▶, 1996 ▶); Dogan *et al.* (1998 ▶).
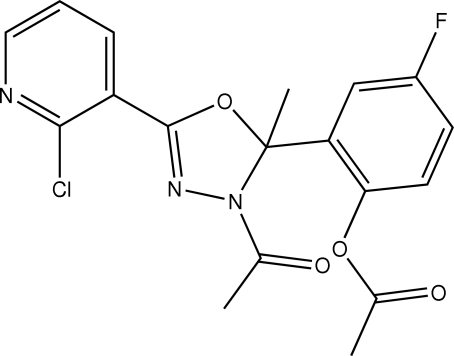


## Experimental

### 

#### Crystal data

C_18_H_15_ClFN_3_O_4_
*M* *_r_* = 391.78
Monoclinic, 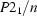

*a* = 10.120 (2) Å
*b* = 13.900 (3) Å
*c* = 13.320 (3) Å
β = 102.14 (3)°
*V* = 1831.8 (6) Å^3^
*Z* = 4
Mo *K*α radiation
μ = 0.25 mm^−1^
*T* = 293 K
0.12 × 0.10 × 0.08 mm

#### Data collection

Bruker APEXII CCD area-detector diffractometer
Absorption correction: multi-scan (*SADABS*; Bruker, 2000 ▶) *T*_min_ = 0.971, *T*_max_ = 0.981
9882 measured reflections
3403 independent reflections
2394 reflections with *I* > 2σ(*I*)
*R*_int_ = 0.026

#### Refinement

*R*[*F* ^2^ > 2σ(*F* ^2^)] = 0.043
*wR*(*F* ^2^) = 0.121
*S* = 1.00
3403 reflections
248 parameters
H-atom parameters not refined
Δρ_max_ = 0.22 e Å^−3^
Δρ_min_ = −0.22 e Å^−3^

### 

Data collection: *APEX2* (Bruker, 2000 ▶); cell refinement: *SAINT* (Bruker, 2000 ▶); data reduction: *SAINT*; program(s) used to solve structure: *SHELXS97* (Sheldrick, 2008 ▶); program(s) used to refine structure: *SHELXL97* (Sheldrick, 2008 ▶); molecular graphics: *SHELXTL* (Sheldrick, 2008 ▶); software used to prepare material for publication: *SHELXTL*.

## Supplementary Material

Crystal structure: contains datablocks I, global. DOI: 10.1107/S1600536809015323/tk2411sup1.cif

Structure factors: contains datablocks I. DOI: 10.1107/S1600536809015323/tk2411Isup2.hkl

Additional supplementary materials:  crystallographic information; 3D view; checkCIF report

## Figures and Tables

**Fig. 1.**
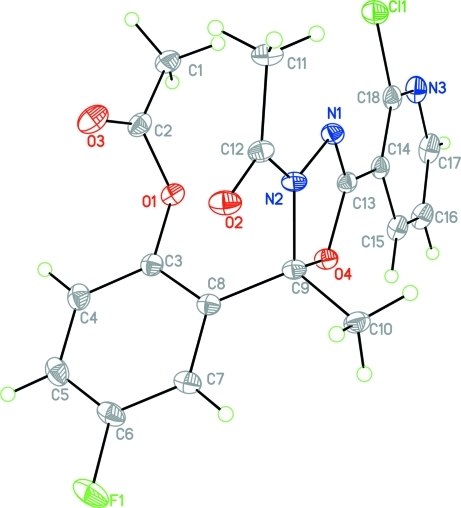
View of (I) showing the atom-labelling scheme. Displacement ellipsoids are drawn at the 40% probability level. H atoms are represented by circles of arbitrary size.

## supplementary crystallographic information

### Comment

In continuation of our study of 1,3,4-oxadiazoline derivatives (Song *et al.*, 2006a,b; Pan *et al.*, 2007), which possess a wide range of pharmaceutical activities (Chimirri *et al.*, 1994, 1996; Dogan *et al.*, 1998), a series of new 1,3,4-oxadiazoline derivatives have been prepared. We present herein the crystal structure of the title compound, (I).

In (I), Fig. 1, the molecule is twisted about the C8—C9 bond. Within the five-membered oxadiazoline ring, there is a formal C13===N1 double bond (1.282 (3) Å). The bond distance of C13—O4 (1.360 (2) Å) is considerably shorter than the of C9—O4 bond (1.452 (2) Å), suggesting some delocalization of π-electron density over the O4—C13—N1 chromophore.

### Experimental

A solution of 2-chloro-*N*'-(1-(5-fluoro-2-hydroxyphenyl)ethylidene) nicotinohydrazide (0.5 g, 1.62 mmol) in acetic anhydride (10 ml) was refluxed until the reaction was finished. The acetic anhydride was distilled under vacuum. The residue was recrystallized from ethanol (10 ml). Colorless crystals (0.46 g) were obtained by slow evaporation of an ethanol solution of (I) after 2 days at room temperature.

### Refinement

All H atoms were placed in calculated positions and allowed to ride on their parent atoms, with C—H = 0.93–0.96 Å and refined in the riding model approximation with *U*_iso_(H) = 1.2—1.5*U*_eq_(C).

### Crystal data

**Table:**

C_18_H_15_ClFN_3_O_4_	*F*(000) = 808
*M**_r_* = 391.78	*D*_x_ = 1.421 Mg m^−^^3^
Monoclinic, *P*2_1_/*n*	Mo *K*α radiation, λ = 0.71073 Å
Hall symbol: -P 2yn	Cell parameters from 3403 reflections
*a* = 10.120 (2) Å	θ = 2.1–25.5°
*b* = 13.900 (3) Å	µ = 0.25 mm^−^^1^
*c* = 13.320 (3) Å	*T* = 293 K
β = 102.14 (3)°	Block, colorless
*V* = 1831.8 (6) Å^3^	0.12 × 0.10 × 0.08 mm
*Z* = 4	

### Data collection

**Table:**

Bruker APEXII CCD area-detector diffractometer	3403 independent reflections
Radiation source: fine-focus sealed tube	2394 reflections with *I* > 2σ(*I*)
graphite	*R*_int_ = 0.026
φ and ω scans	θ_max_ = 25.5°, θ_min_ = 2.1°
Absorption correction: multi-scan (*SADABS*; Bruker, 2000)	*h* = −12→7
*T*_min_ = 0.971, *T*_max_ = 0.981	*k* = −16→16
9882 measured reflections	*l* = −16→16

### Refinement

**Table:**

Refinement on *F*^2^	Primary atom site location: structure-invariant direct methods
Least-squares matrix: full	Secondary atom site location: difference Fourier map
*R*[*F*^2^ > 2σ(*F*^2^)] = 0.043	Hydrogen site location: inferred from neighbouring sites
*wR*(*F*^2^) = 0.121	H-atom parameters not refined
*S* = 1.00	*w* = 1/[σ^2^(*F*_o_^2^) + (0.06*P*)^2^ + 0.3383*P*] where *P* = (*F*_o_^2^ + 2*F*_c_^2^)/3
3403 reflections	(Δ/σ)_max_ < 0.001
248 parameters	Δρ_max_ = 0.22 e Å^−^^3^
0 restraints	Δρ_min_ = −0.22 e Å^−^^3^

### Special details

**Table:**

Geometry. All e.s.d.'s (except the e.s.d. in the dihedral angle between two l.s. planes) are estimated using the full covariance matrix. The cell e.s.d.'s are taken into account individually in the estimation of e.s.d.'s in distances, angles and torsion angles; correlations between e.s.d.'s in cell parameters are only used when they are defined by crystal symmetry. An approximate (isotropic) treatment of cell e.s.d.'s is used for estimating e.s.d.'s involving l.s. planes.
Refinement. Refinement of *F*^2^ against ALL reflections. The weighted *R*-factor *wR* and goodness of fit *S* are based on *F*^2^, conventional *R*-factors *R* are based on *F*, with *F* set to zero for negative *F*^2^. The threshold expression of *F*^2^ > σ(*F*^2^) is used only for calculating *R*-factors(gt) *etc*. and is not relevant to the choice of reflections for refinement. *R*-factors based on *F*^2^ are statistically about twice as large as those based on *F*, and *R*- factors based on ALL data will be even larger.

### Fractional atomic coordinates and isotropic or equivalent isotropic displacement parameters (Å^2^)

**Table:**

	*x*	*y*	*z*	*U*_iso_*/*U*_eq_	
C1	0.0490 (3)	0.1369 (2)	0.5790 (2)	0.0842 (8)	
H1A	−0.0049	0.1943	0.5694	0.126*	
H1B	0.0010	0.0859	0.5383	0.126*	
H1C	0.1328	0.1485	0.5580	0.126*	
C2	0.0772 (2)	0.10902 (17)	0.6890 (2)	0.0686 (6)	
C3	0.1358 (2)	−0.02657 (15)	0.79837 (16)	0.0566 (5)	
C4	0.0293 (3)	−0.04387 (16)	0.8454 (2)	0.0702 (7)	
H4	−0.0576	−0.0247	0.8140	0.084*	
C5	0.0520 (3)	−0.08956 (18)	0.9390 (2)	0.0778 (7)	
H5	−0.0187	−0.1021	0.9718	0.093*	
C6	0.1815 (3)	−0.11600 (17)	0.98264 (17)	0.0729 (7)	
C7	0.2892 (3)	−0.09887 (15)	0.93740 (16)	0.0627 (6)	
H7	0.3758	−0.1177	0.9699	0.075*	
C8	0.2675 (2)	−0.05317 (14)	0.84270 (15)	0.0523 (5)	
C9	0.3835 (2)	−0.03837 (14)	0.78801 (14)	0.0517 (5)	
C10	0.5212 (2)	−0.07353 (16)	0.84381 (18)	0.0655 (6)	
H10A	0.5183	−0.1419	0.8534	0.098*	
H10B	0.5454	−0.0425	0.9095	0.098*	
H10C	0.5872	−0.0585	0.8039	0.098*	
C11	0.4183 (3)	0.23549 (16)	0.76258 (19)	0.0731 (7)	
H11A	0.3303	0.2645	0.7478	0.110*	
H11B	0.4494	0.2268	0.6999	0.110*	
H11C	0.4801	0.2765	0.8080	0.110*	
C12	0.4110 (2)	0.14036 (15)	0.81247 (16)	0.0551 (5)	
C13	0.35464 (19)	−0.02406 (16)	0.61505 (14)	0.0512 (5)	
C14	0.3289 (2)	−0.06543 (17)	0.51150 (15)	0.0561 (5)	
C15	0.3246 (2)	−0.16524 (19)	0.50291 (18)	0.0692 (6)	
H15	0.3420	−0.2032	0.5617	0.083*	
C16	0.2944 (2)	−0.2080 (2)	0.4075 (2)	0.0806 (8)	
H16	0.2913	−0.2746	0.4009	0.097*	
C17	0.2691 (3)	−0.1500 (3)	0.3232 (2)	0.0896 (9)	
H17	0.2472	−0.1791	0.2590	0.107*	
C18	0.3038 (2)	−0.0137 (2)	0.41987 (16)	0.0655 (6)	
Cl1	0.30866 (7)	0.11042 (5)	0.41760 (5)	0.0845 (3)	
F1	0.2064 (2)	−0.16130 (13)	1.07494 (10)	0.1092 (6)	
N1	0.37927 (17)	0.06330 (13)	0.64394 (12)	0.0543 (4)	
N2	0.39054 (17)	0.06139 (12)	0.75037 (12)	0.0525 (4)	
N3	0.2739 (2)	−0.0537 (2)	0.32705 (14)	0.0793 (6)	
O1	0.10784 (15)	0.01308 (10)	0.69914 (11)	0.0612 (4)	
O2	0.42063 (17)	0.13026 (11)	0.90474 (11)	0.0697 (5)	
O3	0.0761 (2)	0.15939 (13)	0.76138 (16)	0.0960 (6)	
O4	0.34983 (15)	−0.08897 (10)	0.69061 (10)	0.0569 (4)	

### Atomic displacement parameters (Å^2^)

**Table:**

	*U*^11^	*U*^22^	*U*^33^	*U*^12^	*U*^13^	*U*^23^
C1	0.0707 (17)	0.0814 (18)	0.097 (2)	0.0185 (13)	0.0106 (14)	0.0250 (15)
C2	0.0516 (13)	0.0587 (15)	0.0921 (19)	0.0046 (11)	0.0072 (12)	−0.0006 (14)
C3	0.0683 (15)	0.0453 (11)	0.0562 (12)	−0.0010 (10)	0.0132 (11)	−0.0100 (9)
C4	0.0750 (16)	0.0595 (15)	0.0802 (17)	−0.0053 (12)	0.0258 (13)	−0.0181 (12)
C5	0.098 (2)	0.0677 (16)	0.0785 (18)	−0.0171 (15)	0.0424 (16)	−0.0228 (14)
C6	0.116 (2)	0.0610 (15)	0.0452 (13)	−0.0158 (14)	0.0260 (14)	−0.0094 (10)
C7	0.0875 (17)	0.0523 (13)	0.0470 (12)	−0.0097 (11)	0.0112 (12)	−0.0046 (10)
C8	0.0686 (14)	0.0409 (11)	0.0462 (11)	−0.0038 (9)	0.0097 (10)	−0.0070 (8)
C9	0.0636 (13)	0.0448 (11)	0.0434 (11)	0.0005 (9)	0.0036 (9)	0.0021 (8)
C10	0.0662 (14)	0.0631 (14)	0.0630 (14)	0.0084 (11)	0.0038 (11)	0.0152 (11)
C11	0.0900 (18)	0.0493 (13)	0.0749 (16)	−0.0044 (12)	0.0058 (13)	0.0103 (11)
C12	0.0573 (13)	0.0493 (12)	0.0531 (13)	0.0000 (9)	−0.0012 (10)	0.0032 (9)
C13	0.0449 (11)	0.0609 (13)	0.0457 (11)	0.0075 (10)	0.0048 (9)	0.0037 (10)
C14	0.0425 (11)	0.0771 (15)	0.0474 (12)	0.0085 (10)	0.0065 (9)	−0.0007 (10)
C15	0.0611 (14)	0.0862 (18)	0.0574 (14)	0.0141 (12)	0.0058 (11)	−0.0118 (12)
C16	0.0746 (17)	0.095 (2)	0.0675 (17)	0.0166 (14)	0.0046 (13)	−0.0188 (14)
C17	0.0727 (18)	0.132 (3)	0.0597 (17)	0.0181 (18)	0.0046 (13)	−0.0288 (17)
C18	0.0463 (12)	0.0983 (18)	0.0509 (13)	0.0108 (11)	0.0077 (10)	0.0046 (12)
Cl1	0.0910 (5)	0.0999 (5)	0.0607 (4)	0.0095 (4)	0.0117 (3)	0.0222 (3)
F1	0.1671 (18)	0.1102 (13)	0.0567 (9)	−0.0254 (11)	0.0381 (10)	0.0077 (8)
N1	0.0541 (10)	0.0628 (12)	0.0431 (9)	0.0031 (8)	0.0033 (8)	0.0088 (8)
N2	0.0628 (11)	0.0474 (10)	0.0429 (9)	0.0000 (8)	0.0011 (8)	0.0065 (7)
N3	0.0658 (13)	0.123 (2)	0.0473 (12)	0.0129 (13)	0.0071 (9)	−0.0021 (11)
O1	0.0643 (9)	0.0546 (9)	0.0614 (9)	0.0060 (7)	0.0060 (7)	−0.0009 (7)
O2	0.0934 (12)	0.0583 (9)	0.0497 (9)	−0.0047 (8)	−0.0023 (8)	0.0010 (7)
O3	0.1075 (15)	0.0635 (11)	0.1116 (16)	0.0128 (10)	0.0107 (12)	−0.0142 (11)
O4	0.0735 (10)	0.0501 (8)	0.0461 (8)	0.0030 (7)	0.0102 (7)	−0.0016 (6)

### Geometric parameters (Å, °)

**Table:**

C1—C2	1.484 (4)	C10—H10B	0.9600
C1—H1A	0.9600	C10—H10C	0.9600
C1—H1B	0.9600	C11—C12	1.489 (3)
C1—H1C	0.9600	C11—H11A	0.9600
C2—O3	1.194 (3)	C11—H11B	0.9600
C2—O1	1.369 (3)	C11—H11C	0.9600
C3—C4	1.377 (3)	C12—O2	1.220 (2)
C3—C8	1.390 (3)	C12—N2	1.364 (3)
C3—O1	1.405 (2)	C13—N1	1.282 (3)
C4—C5	1.375 (3)	C13—O4	1.360 (2)
C4—H4	0.9300	C13—C14	1.466 (3)
C5—C6	1.367 (4)	C14—C15	1.392 (3)
C5—H5	0.9300	C14—C18	1.393 (3)
C6—F1	1.357 (3)	C15—C16	1.377 (3)
C6—C7	1.373 (3)	C15—H15	0.9300
C7—C8	1.388 (3)	C16—C17	1.362 (4)
C7—H7	0.9300	C16—H16	0.9300
C8—C9	1.520 (3)	C17—N3	1.340 (4)
C9—O4	1.452 (2)	C17—H17	0.9300
C9—N2	1.481 (2)	C18—N3	1.331 (3)
C9—C10	1.516 (3)	C18—Cl1	1.727 (3)
C10—H10A	0.9600	N1—N2	1.398 (2)
			
C2—C1—H1A	109.5	H10A—C10—H10C	109.5
C2—C1—H1B	109.5	H10B—C10—H10C	109.5
H1A—C1—H1B	109.5	C12—C11—H11A	109.5
C2—C1—H1C	109.5	C12—C11—H11B	109.5
H1A—C1—H1C	109.5	H11A—C11—H11B	109.5
H1B—C1—H1C	109.5	C12—C11—H11C	109.5
O3—C2—O1	122.1 (2)	H11A—C11—H11C	109.5
O3—C2—C1	127.7 (2)	H11B—C11—H11C	109.5
O1—C2—C1	110.2 (2)	O2—C12—N2	119.22 (19)
C4—C3—C8	122.2 (2)	O2—C12—C11	123.4 (2)
C4—C3—O1	118.3 (2)	N2—C12—C11	117.36 (19)
C8—C3—O1	119.31 (19)	N1—C13—O4	116.17 (17)
C5—C4—C3	119.7 (3)	N1—C13—C14	129.68 (19)
C5—C4—H4	120.1	O4—C13—C14	114.15 (18)
C3—C4—H4	120.1	C15—C14—C18	116.4 (2)
C6—C5—C4	118.2 (2)	C15—C14—C13	117.68 (19)
C6—C5—H5	120.9	C18—C14—C13	125.8 (2)
C4—C5—H5	120.9	C16—C15—C14	120.2 (2)
F1—C6—C5	119.3 (3)	C16—C15—H15	119.9
F1—C6—C7	117.7 (3)	C14—C15—H15	119.9
C5—C6—C7	123.0 (2)	C17—C16—C15	118.2 (3)
C6—C7—C8	119.4 (2)	C17—C16—H16	120.9
C6—C7—H7	120.3	C15—C16—H16	120.9
C8—C7—H7	120.3	N3—C17—C16	124.1 (2)
C7—C8—C3	117.5 (2)	N3—C17—H17	118.0
C7—C8—C9	120.5 (2)	C16—C17—H17	118.0
C3—C8—C9	121.92 (18)	N3—C18—C14	124.3 (3)
O4—C9—N2	99.78 (14)	N3—C18—Cl1	113.72 (19)
O4—C9—C10	107.50 (17)	C14—C18—Cl1	122.02 (18)
N2—C9—C10	111.29 (17)	C13—N1—N2	104.80 (16)
O4—C9—C8	107.64 (16)	C12—N2—N1	124.70 (17)
N2—C9—C8	112.70 (16)	C12—N2—C9	124.06 (16)
C10—C9—C8	116.38 (17)	N1—N2—C9	111.19 (15)
C9—C10—H10A	109.5	C18—N3—C17	116.8 (2)
C9—C10—H10B	109.5	C2—O1—C3	118.16 (18)
H10A—C10—H10B	109.5	C13—O4—C9	107.56 (15)
C9—C10—H10C	109.5		
			
C8—C3—C4—C5	−0.5 (3)	C15—C14—C18—Cl1	−178.79 (16)
O1—C3—C4—C5	174.88 (19)	C13—C14—C18—Cl1	3.5 (3)
C3—C4—C5—C6	0.3 (3)	O4—C13—N1—N2	−1.3 (2)
C4—C5—C6—F1	−179.9 (2)	C14—C13—N1—N2	177.94 (19)
C4—C5—C6—C7	0.1 (4)	O2—C12—N2—N1	−179.27 (18)
F1—C6—C7—C8	179.65 (18)	C11—C12—N2—N1	1.6 (3)
C5—C6—C7—C8	−0.4 (3)	O2—C12—N2—C9	−2.1 (3)
C6—C7—C8—C3	0.2 (3)	C11—C12—N2—C9	178.74 (19)
C6—C7—C8—C9	−176.51 (19)	C13—N1—N2—C12	−177.06 (19)
C4—C3—C8—C7	0.2 (3)	C13—N1—N2—C9	5.5 (2)
O1—C3—C8—C7	−175.12 (17)	O4—C9—N2—C12	175.46 (18)
C4—C3—C8—C9	176.90 (19)	C10—C9—N2—C12	−71.3 (3)
O1—C3—C8—C9	1.5 (3)	C8—C9—N2—C12	61.5 (2)
C7—C8—C9—O4	119.08 (18)	O4—C9—N2—N1	−7.0 (2)
C3—C8—C9—O4	−57.5 (2)	C10—C9—N2—N1	106.21 (19)
C7—C8—C9—N2	−131.88 (18)	C8—C9—N2—N1	−120.95 (17)
C3—C8—C9—N2	51.6 (2)	C14—C18—N3—C17	−0.5 (3)
C7—C8—C9—C10	−1.6 (3)	Cl1—C18—N3—C17	179.67 (18)
C3—C8—C9—C10	−178.13 (18)	C16—C17—N3—C18	−0.8 (4)
N1—C13—C14—C15	170.8 (2)	O3—C2—O1—C3	1.4 (3)
O4—C13—C14—C15	−10.0 (3)	C1—C2—O1—C3	−179.18 (19)
N1—C13—C14—C18	−11.5 (3)	C4—C3—O1—C2	75.7 (2)
O4—C13—C14—C18	167.70 (19)	C8—C3—O1—C2	−108.7 (2)
C18—C14—C15—C16	−1.0 (3)	N1—C13—O4—C9	−3.4 (2)
C13—C14—C15—C16	176.9 (2)	C14—C13—O4—C9	177.29 (16)
C14—C15—C16—C17	−0.1 (4)	N2—C9—O4—C13	5.97 (19)
C15—C16—C17—N3	1.1 (4)	C10—C9—O4—C13	−110.18 (18)
C15—C14—C18—N3	1.4 (3)	C8—C9—O4—C13	123.72 (16)
C13—C14—C18—N3	−176.3 (2)		
